# Oral status and affecting factors in Iranian ICU patients: a cross-sectional study

**DOI:** 10.1186/s12903-023-02867-6

**Published:** 2023-03-17

**Authors:** Mostafa Arkia, Jahangir Rezaei, Nader Salari, Siavash Vaziri, Alireza Abdi

**Affiliations:** 1 Student Research Committee, Nursing and Midwifery School, Kermanshah University of Medical Sciences, Kermanshah, Iran; 2 Department of Medical and Surgical Nursing, Nursing and Midwifery School, Kermanshah University of Medical Sciences, Kermanshah, Iran; 3 Department of Biostatistics, School of Health, Medical Biology Research Center, Kermanshah University of Medical Sciences, Kermanshah, Iran; 4 Department of Infectious Disease, School of Medicine, Kermanshah University of Medical Sciences, Kermanshah, Iran; 5 Department of Emergency and Critical Care Nursing, Nursing and Midwifery School, Kermanshah University of Medical Sciences, Kermanshah, Iran

**Keywords:** Intensive care unit, Oral care, Nursing, Nurse

## Abstract

**Background:**

Oral care is crucial in intensive care units (ICUs). Meanwhile, this action is not well-performed, therefore, mouth cavity-associated disorders cause serious outcomes, e.g. ventilator-dependent pneumonia. Considering a lack of studies in Iran on this subject, this study aimed to determine the oral status and affected factors in ICU patients in Iran.

**Methods:**

In a cross-sectional study in 2019, we assessed the oral status of 138 patients admitted to the ICUs in the Kermanshah and Ilam provinces by census method. The tools were a demographic and clinical characteristics checklist, and Beck's oral status assessment scale (BOAS). The researcher investigated the condition of the patient's mouth, and their records. Data were analyzed using descriptive and inferential statistics.

**Results:**

In this study, the prevalence of moderate and severe disorders of the lips, gums and oral mucosa, tongue, teeth, and saliva were 14.4, 26.1, 16.6, 49.3, and 34.8 percent, respectively. Six percent of patients had a normal oral condition. Oral status had a significant relationship with education level, age, marital status, brushing teeth, NG tube, and consciousness level.

**Conclusion:**

Compared to other studies, the prevalence of oral cavity disorders in ICU patients of the Ilam and Kermanshah provinces was high. It mandates paying crucial attention to nurses' continued education, using standard guidelines, and applying new facilities. Moreover, it is mandated for periodical visits of patients by a dentist in ICUs.

## Background

In the intensive care unit (ICU), critically ill patients are continuously monitored and cared for while providing specialized services [1]. Statistics show that 62.2% of ICU patients undergo complications during hospitalization [2]. They are suffering from oral problems caused by various factors e.g. malnutrition, the presence of endotracheal and stomach tubes, and reduced fluid intake. Studies have shown that inadequate oral care causes further difficulties in dryness of the oral mucosa, decrease in saliva flow, inflammation of the oral mucosa, formation of dental plaque, inflammation of the gums, and accumulation of pathogenic bacteria in the mouth and throat [3]. Bacteria present in dental plaques create heart infections, joint diseases, and ventilator-dependent pneumonia (VAP). As the most common and dangerous hospital infection in ICUs, VAP causes 50% mortality [4].

The indecent condition of the mouth induces the natural flora of the mouth to change in favor of negative microorganisms. Therefore, in these patients, oral fibronectin decreases. Likewise, increasing the strength of bacteria to attach to the tongue and teeth, then creates oral lesions [5]. It has been proved that proper care of the mouth of patients hospitalized in ICUs can significantly reduce the number of dental plaques, gum diseases, and the occurrence of ventilator-dependent pneumonia [6], however, despite the importance of this topic, researchers have reported that oral care is not performed well in ICUs, procedures are not properly recorded, nursing knowledge in this field is not up-to-date, oral care training for nursing students in colleges is considered less important and there is no standard protocol in this field [3].

In Iran, oral care in patients hospitalized in ICUs is considered ancillary care, so in the study of Ranjber et al., it was found that 55.7% of nurses believed that oral care is the work of the patient's assistant and the patient's family, 9% of nurses believed in not acquiring enough training in the oral care field. Additionally, 69% did not have enough time for oral care, 40% considered oral care unpleasant, and 83.8% of nurses wanted more training about oral care [6]. In Masoumi et al.'s study on the prevalence of oral disorders in patients admitted to the ICUs of a hospital in Zanjan-Iran, the prevalence of oral cavity disorder was 79.7% on the third day and 90.54% on the fourth day of admission [7]. In another study in ICUs of Birjand hospitals in Iran, 84.9% of nurses were trained in oral care, however, oral care was ranked as the 10th priority of nursing care [8]. In the study of Rafael et al., which was conducted under the title of the prevalence of oral disorders in patients hospitalized in ICUs in Brazil, the prevalence of normal structures of the lips, tongue, gums, cheeks, floor of the mouth, and palate was 8.56, 85.4, 85.2, 97.2, 100 and 98.3%, respectively [9].

In developed countries, there are special evaluation systems, guidelines, and protocols for oral cavity evaluation, and it is considered basic care [10, 11]. Though, in Iran and other developing countries this measure is done arbitrarily, without any determined regulations [6].

The prevalence of oral disorders in ICU patients is different in various contexts, based on disparate factors [9]. Due to the lack of studies in Iran regarding oral cavity disorders in patients hospitalized in ICUs, the present study aimed for determining the rate of oral cavity disorders and affecting factors in ICU patients in the Kermanshah and Ilam provinces, west of Iran.

## Methods

We conducted a descriptive-analytical study from January to June 2019 in ICU wards of educational hospitals affiliated with Kermanshah and Ilam Universities of Medical Sciences. The sampling method was census. Herewith, we recruited all patients (with any condition) who met the inclusion criteria, 138 patients in total. People aged over 18 years, hospitalized in ICUs for at least 48 h, and absent of oral lesions or trauma once admitted were enrolled. We exclude the patients with incomplete information in their records and no consent to participate in the study. The tool had three elements; the demographic characteristics form including gender, job, educational level, marital status, age, income, and height; the clinical checklist consists of the person in charge of mouth care, using a toothbrush, materials used for mouth washing, using ointment, GCS, total duration of intubation, number of mouth wash per day, diet method, received food type, and having NG-tube; and the standard scale for examining oral cavity status entitled Beck Oral Assessment Scale (BOAS) [7].

**Fig. 1:**
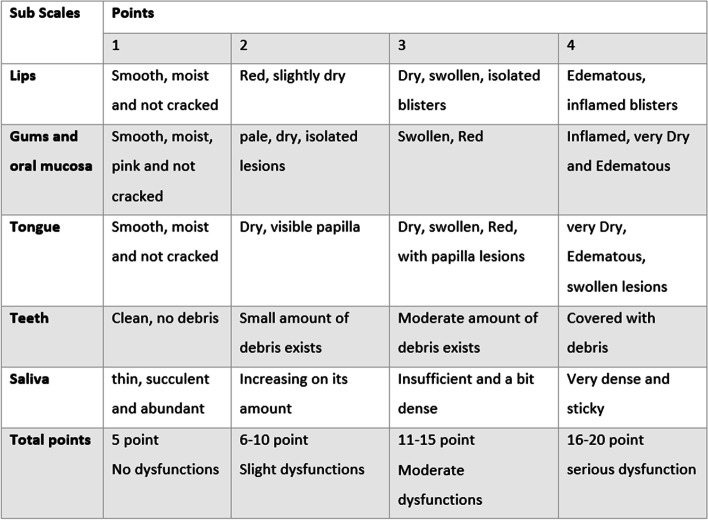
The components of Beck Oral Assessment Scale

Beck Oral Assessment Scale has five sub-scales that evaluate lips, mucous and gums, teeth, saliva, and tongue, and each is graded in four points from 1 to 4. The overall score is between 5 and 20. The highest the score, the more severe of oral disorder. Likewise, a score of 5 indicates no disorder, 6–10 mild disorder, 11–15 moderate disorder, and 16–20 severe disorder [7]. It was designed and validated by Beck in 1974 [12]. Figure 1 shows the components of BOAS, which is brought from the study of Nguh (2016) [12].

Safarabadi et al. (2017) confirmed the validity and reliability of the BOAS tool, in which content validity was supported by three anesthesiologists, two neurosurgeons, and five expert nurses working in ICUs. The test–retest method has been used to determine its reliability by evaluating twenty ICU patients by two observers separately, and a correlation coefficient of 0.92 was obtained [13]. For the demographic form and the clinical checklist, ten academic members and experts in the ICU area approved the content validity of the checklists, and their opinions were considered.

Data collection was begun after obtaining permission from the vice-chancellor of research and technology of Ilam and Kermanshah Universities of Medical Sciences and presenting it to the officials of the selected hospitals, which were in Kermanshah (Imam Reza, Imam Ali, Taleghani, Imam Khomeini and Farabi hospitals) and Ilam (Imam Khomeini and Shahid Mustafa Khomeini hospitals) cities. After introducing and commenting on the research objectives and obtaining informed consent from the research samples or their legal guardians, the first researcher collected the data. In this way, at first, the ICU clothes were worn, and after entering the ward, the patient's file was checked, and if the patient met the inclusion criteria, clinical and demographic information was recorded. After that, the researcher checked the oral condition after washing his hands for 30 s with soap and water and wearing disposable gloves, a mask, and glasses. Herewith, first, the lips were observed and then the patient's mouth opened slowly. With the use of a tongue depressor stick and a flashlight, the patient's mouth status was observed and recorded in the questionnaire.

Data were analyzed using descriptive statistics and non-parametric tests such as Mann–Whitney U and Kruskal–Wallis and Spearman's correlation by SPSS-25 software. The significant level of all tests was less than 0.05.

## Results

In this study, 138 ICU patients were recruited, 67.4% of them were male, 20% were unemployed, 25.4% illiterate, and 85.5% lived in the city (Table 1). The mean and SD of age, income, and height were 57.68 ± 18.44 years, 15.50 ± 12.30 million Rial (Iran currency), and 169.18 ± 8.24 cm, respectively. In six percent of patients, the general status of the mouth was normal and 94% had at least one problem in one of the components. Moderate and severe disorders of the lip, gum, and oral mucosa structures, teeth, and saliva were 14.4, 26.1, 16.6, 49.3 and 34.8%, respectively (Table 2).

**Table 1:** Frequency and frequency percentage of demographic variables

Variable	Frequency(%)
Job	
Self-employment	43(%31)
Employed	6(%4.3)
Retired	29(%21)
Unemployed	28(%20)
Other	32(%23.2)
Total	138(%100)
Location	
Rural	20(%14.5)
Urban	118(%85.5)
Total	138(%100)
Gender	
Male	93(%67.4)
Female	45(%32.6)
Total	138(%100)
Marital status	
Single	15(%10.9)
Married	110(%79.7)
Widow/divorced	13(%9.4)
Total	138(%100)
Education level	
Illiterate	35(%25.4)
Under diploma	61(%44.2)
Diploma	37(%26.8)
Academic	5(%3.6)
Total	138(%100)
Person in charge of mouth care	
Nurse	120 (87.0)
Nurse assistance	4 (2.9)
Other	14 (10.1)
Tooth brushing	
Yes	23 (16.7)
No	115 (83.3)
Material used for mouth washing	
Chlorhexidine	111 (80.5)
Normal saline	18 (13)
Other	9 (6.5)
Use of emollient ointment	
Yes	79 (57.2)
No	59 (42.8)

**Table 2:** Descriptive data on the prevalence of oral status

Variable	Level of disorder	Frequency(%)
Lips	normal	64(%46.4)
	mild	54(%39.1)
	Moderate	10(%7.2)
	sever	10(%7.2)
	total	138(%100)
Gum and oral mucosa	normal	49(%35.5)
	mild	53(%38.4)
	Moderate	31(%32.5)
	sever	5(%3.6)
	total	138(%100)
Language	normal	54(%39.1)
	mild	61(%44.2)
	Moderate	21(%15.2)
	sever	2(%1.4)
	total	138(%100)
Teeth	normal	19(%13.8)
	mild	41(%29.7)
	Moderate	57(%41.3)
	sever	11(%8)
	total	138(%100)
Saliva	normal	63(%45.7)
	mild	27(%19.6)
	Moderate	44(%31.9)
	sever	4(%2.9)
	total	138(%100)
Overall oral status	normal	8(%5.8)
	mild	64(%46.4)
	Moderate	50(%36.2)
	sever	7(%5.1)
	total	138(%100)

Inferential statistics revealed that people who either had a university education (*p* < 0.01), or married patients (*p* = 0.03), had better oral condition, however, the problem was worse in older patients (*p* = 0.009). Brushing teeth (*p* < 0.001) in nursing care was accompanied by a lower BOAS score. By reducing the level of consciousness the oral status would be more compromised (*p* = 0.039). No significant relationship was found between the number of daily mouth washing, gender, duration of intubation, type of mouthwash, and the person in charge of mouth washing with the overall score of mouth status (Tables 3, 4 and 5). The overall oral status score was higher in patients with a Naso-gastro tube (NG tube)(Tables 6 and 7).

**Table 3:** Relationship of oral status variables with qualitative demographic and clinical variables

Oral status variables		Saliva status score		tongue status score		Teeth status	
Demographic and clinical variables		Mean(SD)	Mean rank	Mean(SD)	Mean rank	Mean(SD)	Mean rank
Gender	Male	1.94(0.95)	70.41	1.84(0.75)	71.90	2.29(1.03)	69.21
	female	1.86(0.91)	67.42	1.69(0.73)	64.54	2.29(1.05)	70.1
Statistical analysis (Mann–Whitney U test)		Z = -0.41 *p* = 0.68		Z = -1.09 *p* = 0.27		Z = -0.12 *P* = 0.89	
Job	Self-employment	1.88(0.85)	68.85	2.3(1.103)	80.09	2.3(1.1)	69.81
	employed	2.16(0.98)	79.75	2(0.89)	56.25	2(0.89)	56.33
	retired	2.1(1/11)	75.22	2.55(0.73)	66.12	2.55(0.79)	78.36
	unemployed	1.53(0.83)	54.07	2.18(1.18)	67.68	2.18(1.18)	65.3
	other	2.09(0.93)	76.77	2.19(1.09)	62.41	2.19(1.09)	67.19
Statistical analysis (Kruskal–Wallis test)		K2 = 7.19 *P* = 0.12		K2 = 5.82 *p* = 0.21		K2 = 5.82 *p* = 0.21	
Location	rural	2(0.85)	73.68	1.75(0.73)	68.15	2.2(1.15)	68.03
	urban	1.9(0.96)	68.79	1.8(0.75)	69.73	2.31(1.02)	69.75
Statistical analysis (Mann–Whitney U test)		Z = -0.54 *p* = 0.58		Z = -0.17 *p* = 0.85		Z = -0.18 *p* = 0.85	
Marital status	single	1.4(0.63)	49.37	1.93(1.28)	58.8	1.93(1.28)	54.09
	married	1.95(0.96)	70.74	2.3(1)	71.75	2.3(1)	70.02
	Widow/divorced	2.23(0.92)	82.27	2.62(1.04)	61.62	2.62(1.04)	81.96
Statistical analysis (Kruskal–Wallis test)		K2 = 6.05 *p* = 0.04*		K2 = 2.04 *p* = 0.36		K2 = 3.64 *p* = 0.16	
Level of education	illiterate	1.97(0.92)	71.91	2.29(1.15)	69.97	2.29(1.15)	71.78
	Under diploma	2.11(0.95)	77.2	2.51(1.04)	79.84	2.51(1.4)	78.5
	diploma	1.54(0.86)	54.01	1.92(0.89)	53.03	1.92(0.89)	52.41
	Academic	2(1)	73.2	2.4(0.54)	62	2.4(0.54)	69.6
Statistical analysis (Kruskal–Wallis test)		K2 = 9.23 *p* = 0.02*		K2 = 12.39 *p* = 0.006*		K2 = 11.08 *p* = 0.01*	
Person in charge of mouth care	nurse	1.93(0.83)	68.64	1.76(0.85)	70.51	9.94(3.27)	66.01
	Nurse assistance	1.9(0.73)	69.1	1.3(0.48)	50.20	8.5(3.34)	49.25
	other	2.11(1.02)	75	1.94(1.11)	74.06	10.12(4.33)	64.38
Statistical analysis (Kruskal–Wallis test)		K2 = 0.44 *p* = 0.8		K2 = 3.13 *p* = 0.2		K2 = 1.88 *p* = 0.39	
Tooth brushing	yes	1.43(0.66)	46.39	1.17(0.49)	41.59	7.56(2.77)	38.39
	no	2(0.85)	74.12	1.87(0.89)	75.08	10.35(3.35)	70.22
Statistical analysis (Mann–Whitney U test)		Z = -3.22 *P* < 0.001*		Z = -4 *P* < 0.001*		Z = -3.78 *P* < 0.001*	
Material used for mouth washing	chlorhexidine	1.95(0.88)	32.69	1.86(0.91)	73.96	10.04(3.49)	66.61
	Normal saline	1.78(0.64)	63.67	1.28(0.46)	48.49	9(2.93)	56.64
	other	2.22(0.83)	83.33	1.44(0.72)	55.72	9.33(3.57)	58.83
Statistical analysis (Kruskal–Wallis test)		K2 = 1.64 *p* = 0.43		K2 = 8.61 *p* = -0.01*		K2 = 1.33 *p* = 0.52	
Use of emollient ointment	yes	1.95(0.9)	69.43	1.73(0.84)	69.23	9.72(3.59)	63.81
	no	1.93(0.78)	69.53	1.78(0.93)	69.86	9.92(3.3)	65.44
Statistical analysis (Mann–Whitney U test)		Z = -0.56 *p* = 0.57		Z = -0.09 *p* = 0.99		Z = -0.24 *p* = 0.8	

^*^ is significant

**Table 4:** Relationship of oral status variables with demographic and clinical variables

Oral status variable		Gum and oral mucosa status score		Lips status		Overall oral status	
Demographic and clinical variable		Mean(SD)	Mean rank	Mean(SD)	Mean rank	Mean(SD)	Mean rank
Gender	Male	1.95(0.85)	69.77	1.66(0.78)	66.19	9.84(3.29)	64.66
	female	1.93(0.86)	68.93	1.96(1.02)	76.44	9.85(3.69)	64.18
Statistical analysis (Mann–Whitney U test)		Z = -0.12 *p* = 0.9		Z = -1.52 *p* = 0.12		Z = -0.06 *p* = 0.94	
Job	Self-employee	2.07(0.82)	75.51	1.77(0.89)	69.97	10.28(3.33)	69.49
	employee	1.5(0.83)	49	1.83(0.4)	81.67	9(2.75)	55.5
	retired	2(0.92)	71.76	1.59(0.78)	62.60	10.10(3.48)	68.61
	unemployed	1.93(0.97)	67.43	1.68(1.02)	62.46	9.14(4)	54.59
	other	1.81(0.69)	65.03	1.94(0.87)	79	9.85(3.05)	65.93
Statistical analysis (Kruskal–Wallis test)		K2 = 3.51 *p* = 0.47		K2 = 4.88 *p* = 0.3		K2 = 3.25 *p* = 0.51	
Location	rural	2(0.85)	72.10	2(0.91)	20.81	10.58(3.6)	72.12
	urban	1.93(0.85)	69.06	1.71(0.86)	53/67	9.73(3.39)	62.33
Statistical analysis (Mann–Whitney U test)		Z = -0.33 *p* = 0.73		Z = -1.54 *p* = 0.12		Z = -0.91 *p* = 0.36	
Marital status	single	1.8(0.94)	62.8	0.91(0.21)	57.10	8(3.21)	43.07
	married	1.96(0.84)	70.57	0.83(0.08)	69.05	8.96(3.37)	65.65
	Widow/divorced	1.92(0.86)	68.15	1.09(0.03)	87.58	11.08(3.44)	79.75
Statistical analysis (Kruskal–Wallis test)		K2 = 0.58 *p* = 0.74		K2 = 4.9 *p* = 0.08		K2 = 6.87 *p* = 0.03*	
Level of education	illiterate	2.06(0.87)	74.76	1.77(0.8)	73.13	10.46(2.83)	73.15
	Under diploma	2.11(0.87)	77.13	1.93(0.99)	75.81	10.83(3.75)	74.4
	diploma	1.54(0.67)	51.84	1.49(0.69)	58.42	7.91(2.56)	44.88
	academic	2(1)	72.40	1.4(0.54)	56.10	9.4(2.96)	61.7
Statistical analysis (Kruskal–Wallis test)		K2 = 11.27 *p* = 0.01*		K2 = 6.04 *p* = 0.1		K2 = 18.42 *P* < 0.03*	
Person in charge of mouth care	nurse	1.93(0.83)	68.64	1.84(0.85)	70.51	9.94(3.27)	66.01
	Nurse assistance	1.9(0.73)	69.10	1.3(0.48)	50.20	9.94(3.27)	44.25
	other	2.11(1.02)	75	1.94(1.11)	74.06	10.12(4.33)	64.38
Statistical analysis (Kruskal–Wallis test)		K2 = 0.44 *p* = 0.8		K2 = 3.13 *p* = 0.2		K2 = 1.88 *p* = 0.39	
Tooth brushing	yes	1.43(0.66)	46.39	1.17(0.49)	41.59	7.56(2.77)	38.39
	no	2(0.85)	74.12	1.87(0.89)	75.08	10.35(3.35)	70.22
Statistical analysis (Mann–Whitney U test)		Z = -3.22 *p* = 0.001*		Z = -4 *P* < 0.001*		Z = -3.78 *P* < 0.001*	
Material used for mouth washing	chlorhexidine	1.95(0.88)	32.69	1.86(0.91)	73.96	10.04(3.49)	66.41
	Normal saline	1.78(0.64)	63.67	1.28(0.46)	48.49	9(2.93)	56.64
	other	2.22(0.83)	83.13	1.44(0.72)	55.72	9.33(3.57)	58.83
Statistical analysis (Kruskal–Wallis test)		K2 = 1.64 *p* = 0.43		K2 = 8.61 *p* = 0.01*		K2 = 1.3 *p* = 0/52	
Use of emollient ointment	yes	1.95(0.9)	69.43	1.73(0.84)	69.23	9.79(3.52)	63.81
	no	1.93(0.78)	69.53	1.78(0.93)	69.86	9.92(3.3)	65.44
Statistical analysis (Mann–Whitney U test)		Z = -0.56 *p* = 0.57		Z = -0.09 *p* = 0.99		Z = -0.24 *p* = 0.8	

^*^ is significant

**Table 5:** Correlation between oral variables with quantitative demographic and clinical variables by Spearman's correlation

Oral position variable	Saliva status	Teeth status	tongue status	Gum and oral mucosa status	Lips status	Overall oral statuse
Demographic and clinical variable						
Age(years) Mean (57.68)	*r* = 0.184 *p* = 0.031*	*r* = 0.079 *p* = 0.0355	*r* = 0.08 *p* = 0.352	*r* = 0.059 *p* = 0.488	*r *= 0.143 *p* = 0.095	*r* = 0.229 *p* = 0.009*
Income(million toman) mean(1.55 toman)	*r *= 0.106 *p* = 0.216	*r* = 0.074 *p* = 0.375	*r* = -0.005 *p* = 0.953	*r* = 0.006 *p* = 0.948	*r* = -0.076 *p* = 0.374	*r* = 0.059 *p* = 0.507
Height(centimeter) Mean (169.18)	*r* = 0.109 *p* = 0.203	*r* = 0.029 *p* = 0.736	*r* = 0.025 *p* = 0.769	*r* = -0.01 *p* = 0.907	*r* = -0.083 *p* = 0.336	*r* = 0.003 *p* = 0.927
GCS(The average of the last 72 horses) Mean (8.76)	*r* = -0.258 *P* = 0.002*	*r* = 0.118* *P* = 0.169	*r* = -0.176 *P* = 0.039*	*r* = -0.138 *P* = 0.106	*r* = -0.243 *P* = 0.004*	*r* = 0.183 *P* = 0.039*
The number of mouthwashes per day Mean (2.71)	*r* = 0.084 *P* = 0.33	*r* = -0.002 *P* = 0.984	*r* = -0.049 *P* = 0.569	*r* = -0.061 *P* = 0.478	*r* = 0.091 *P* = 0.29	*r *= 0.013 *P* = 0.882
Total duration of intubation(day) Mean (7.06)	*r* = 0.019 *P* = 0.822	*r* = -0.068 *P* = 0.426	*r* = 0.025 *P* = 0.768	*r* = -0.02 *P* = 0.819	*r* = -0.16 *P* = 0.062	*r* = 0.094 *P* = 0.291

^*^ is significant

**Table 6:** Relationship of oral status variables with nutritional variables

Oral status variables		Saliva status		tongue status		Teeth status	
Clinical variables		Mean(SD)	Mean rank	Mean(SD)	Mean rank	Mean(SD)	Mean rank
Diet Method	vein	2.16(0.93)	79.75	1.83(0.75)	72.67	11.83(1)	57.13
	gavage	1.93(0.91)	70.49	1.84(73)	72.03	2.27(1)	68.73
	oral	1.42(0.85)	49.25	1.64(73)	60.93	2.21(0.69)	63.21
	other	2(1.03)	71.98	1.73(73)	65.92	2.55(1.06)	78.5
Statistical analysis (Kruskal–Wallis test)		K2 = 5.26 *p* = 0.15		K2 = 1.52 *p* = 0.47		K2 = 3.55 *P* = 0.31	
Food type	Intralipid	1.75(0.96)	62.58	1.67(0.65)	64.46	22.25(0.96)	68.29
	Homemade food	1.66(1.07)	57.88	1.75(0.74)	67.88	1.92(1.16)	58
	Food package	1.98(0.89)	72.52	1.91(0.8)	75.04	2.29(0.99)	70.02
	Hospital food	1.5(0.84)	52.6	1.5(0.7)	54.6	2(0.94)	54.2
	NPO	2.08(1.01)	75.41	1.78(0.75)	69.09	2.51(1.12)	77.78
	other	1.91(0.9)	70.08	1.67(0.65)	64.46	2.25(1.05)	67.04
Statistical analysis (Kruskal–Wallis test)		K2 = 4.95 *P* = 0.42		K2 = 3.35 *p* = 0.64		K2 = 4.56 *p* = 0.47	
NG-Tube	yes	2.01(0.44)	73.41	1.82(0.74)	70.94	2.23(1.07)	71.23
	No	1.27(0.66)	43.44	1.62(0.74)	59.85	2.06(0.8)	58
Statistical analysis (Mann–Whitney U test)		Z = -3.18 *p = 0.001		Z = -1.18 *p* = 0.23		Z = -1.38 *p* = 0.16	

^*^ is significant

**Table 7:** Relationship of oral status variables with nutritional variables

Oral status variables		Gum and oral mucosa status		Lips status		Overall oral status	
Clinical variables		Mean(SD)	Mean rank	Mean(SD)	Mean rank	Mean(SD)	Mean rank
Diet Method	vein	2(0.95)	72.25	2.08(0.79)	87.83	9.88(2.84)	65.44
	gavage	1.94(0.86)	68.94	1.85(0.93)	73.07	10.13(3.55)	67.72
	oral	1.93(0.61)	71.07	1.21(0.42)	45.14	8.35(2.56)	84.96
	other	1.94(0.89)	69.18	1.64(0.82)	64.62	9.84(3.53)	63.69
Statistical analysis (Kruskal–Wallis test)		K2 = 0.1 *p* = 0.99		K2 = 10.52 *p* = 0.01*		K2 = 3.06 *P* = 0.38	
Food type	Intralipid	11.83(0.83)	65.25	1.58(0.51)	66.92	9.36(2.97)	59.91
	Homemade food	1.83(0.57)	66.75	1.58(0.66)	64.67	9.5(3.53)	60.90
	Food package	1.98(0.82)	71.58	1.93(0.97)	75.97	10.37(3.43)	70.81
	Hospital food	1.7(0.94)	56.5	1.2(0.42)	44.30	7.9(3.1)	41.70
	NPO	2.03(0.92)	73.08	1.74(0.89)	69.53	9.97(3.55)	65.29
	other	1.92(0.99)	66.75	1.75(0.96)	68.17	9.63(3.5)	61.32
Statistical analysis (Kruskal–Wallis test)		K2 = 1.97 *P* = 0.85		K2 = 6.73 *p* = 0.24		K2 = 5.67 *p* = 0.33	
NG-Tube	yes	1.97(0.87)	70.38	1.82(0.87)	72.73	10.15(3.44)	67.77
	No	1.78(0.64)	63.67	1.33(0.86)	47.94	8(2.7)	44.50
Statistical analysis (Mann–Whitney U test)		Z = -0.7 *p* = 0.48		Z = 2.67 *p* = 0.007*		Z = -2.48 *p* = 0.001*	

^*^ is significant

## Discussion

The results of this study showed that 14.4% of ICU patients had moderate and severe lip disorder, which is in line with the study conducted by Dakrose et al. in 2014, in which 72 h after admission, 17% of ICU patients had lip ulcers [14]. Lip disorders in ICU patients are affected by the presence of a tracheal tube, tracheal tube fixers, decreasing consciousness level, fever, and dehydration [14, 7]. Moreover, insufficient attention to lip care, improper fixing of the tracheal tube, and inappropriate nursing checklist to assess the lip condition are other reasons.

According to the results of this study, 26.1% of patients had moderate and severe gums and oral mucosa disorders. The results were consistent with the study of Kima et al. in 2019 in South Korea, in which the prevalence of mucosal ulcers in the lower, middle, and upper parts of the mouth was 36.3%, 11.5%, and 7.1%, respectively [15]. The almost high prevalence of gum and oral mucosa lesions in these patients is due to the presence of underlying conditions such as diabetes, endotracheal tube, tracheal tube stabilizers, airway, decreased consciousness, use of sedative drugs, the level of hemoglobin, hematocrit, and low plasma proteins [16]. In addition to the above-mentioned notes, this prevalence could be associated with the continuous opening of the patients' mouths and the remaining dryness of oral mucosa.

According to the results of this study, 16.6% of these patients had moderate and severe tongue disorders. In D'Cruz et al. study in 2014, 72 h after admission to the ICU, 82% of patients had a visible coating on more than 70% of their tongue [14]. Furthermore, this event was intense in non-ICU patients revealed in a descriptive-analytical study by Molania et al. (2018) by examining oral problems in patients referred to behavioral disease counseling centers in Sari city of Iran, and oral lesions prevalence was seen in 96%, and the most common lesions were related to the tongue (80%) [17]. This variation may be related to the difference in medications and nursing care.

In this study, 49.3% of patients had moderate and severe teeth disorders. Masoumi et al. (2015) found the number of oral lesions on the fourth day was 90.54% in ICU patients, and the number of teeth disorders was high and had a direct relationship with VAP [7]. Teeth disorders in ICU patients are disturbances of normal oral flora in these patients. Within 48 h after a person's admission to the hospital, flora changes in favor of Gram-negative organisms with greater pathogenicity. These changes cause the accumulation of bacteria and the proliferation of opportunistic pathogens in the oral cavity and cause local and general complications e.g. stomatitis, tooth decay, infection of the tissues around the tooth, followed by the systematic spread of infection, bacteremia, and respiratory infections such as pneumonia. The infection also affects the joints and the heart [14–20]. In addition, the prevalence of tooth disorders in patients hospitalized in ICUs can be due to nurses' fear of tracheal tubes getting stuck while brushing, and the lack of nurses' training to brush these patients' teeth, which needs more studies.

According to the results of this study, 34.8% of patients had moderate and severe saliva disorders. The causes of salivary disorders include atrophy of salivary glands, use of drugs such as antidepressants, underlying medical conditions, intubation, and the advanced age of most ICU patients [20]. In addition to the above, failure to use mouthwash in the standard way and the lack of proper use of nebulizers for susceptible patients would be considered other sources of saliva disorders.

According to the results of this study, only 5.8% of these patients had normal oral conditions. Inconsistent with the study of Rafael et al. who examined the mouths of patients hospitalized in the ICU department, the results of the oral and dental evaluation showed that the normality of different classes of mouth and palate was 100% and 98.3%, respectively, and 82% of patients did not have any bleeding from the gums [9]. However, other studies showed that patients hospitalized in the ICU have disturbed oral conditions [15, 16]. The high normal condition may be due to the presence of a dental surgeon in the ICUs [9]. Omer et al. (2015) checked the awareness of ICU nurses about the oral care of these patients, it showed that 97.4% of them considered this care important [21]. While in a study conducted in Iran, 55.7% of ICU nurses believed that taking care of the mouth is the job of the nursing assistants and the patient's family [6]. In addition to a lack of knowledge and insufficient training, the high prevalence of oral cavity disorders can be due to the lack of oral and dental specialists in ICUs.

Based on the results of this study, the oral condition was significantly worse in widow patients. The results were inconsistent with the study of Rashidi et al. (2013), which was no significant relationship between oral condition and marital status [22]. Akbari et al. (2014) investigated the oral and dental treatment needs of drug abusers, and the condition of the mouth of divorced people was worse than single or married people [23].

Based on our results, higher education has a positive connection with oral conditions among ICU patients. Ardakani et al. (2013) found education level would play an essential role in predicting the oral and dental health of people. They emphasized that these variables can be changed through training [24]. The possible reason for the worsening of the oral condition with the decrease in the level of education in ICU patients can be connected to less desire and awareness of patients for cooperating with nurses during mouth washing and hygiene.

In this study, older patients had worse mouth conditions. According to the results of Kosari et al.'s study, there was no significant relationship between the indicators related to the condition of the mouth with age [25]. The disparity could be attributed to the difference in population. In general, elderly patients are exposed to various diseases due to physiological and pathological changes; weakness of the immune system, chronic diseases, and the use of many drugs. Moreover, the use of artificial teeth is another factor that affects the oral mucosa in these people [26, 27]. In addition to the mentioned reasons, the prevalence of oral disorders in elderly people in the ICUs can be due to a lack of education, and scientific knowledge of the nurses on specific care of elderly people.

According to the results of this study, the condition of the mouth was better in the patients who brushed their teeth. Shafipour et al. (2015) in a review study declared brushing teeth significantly reduces dental plaques, and the accumulation of oral microorganisms [28], which is in line with our study. Dental plaques are an important source for the growth of microorganisms that cause oral lesions, and brushing is a good way to remove these plaques [28].

According to the results of this study, the condition of the mouth was worse in patients with a low level of consciousness. Masoumi et al. showed there was a significant correlation between the decrease in the level of consciousness and tongue lesions in patients hospitalized in ICUs [7], which was the same as our study. In patients with a decreased level of consciousness due to lack of swallowing and lack of jaw movement, salivary secretions declined, and as a result, the tongue becomes dry .

In the patients of this study who used chlorhexidine to rinse their mouths, their lips were worse than those who applied normal saline. Consistent with our study, Rezaei et al., compared the effect of a mouthwash containing brush wood extract, aloe vera, and chlorhexidine on the gingival index in intubated patients. According to the results, chlorhexidine has side effects such as mucus color change and dryness of the mouth and lips [29].

Our study had some limitation, the first researcher needs to be an expert in assessing oral care by the BOAS scale for data collection, therefore, he was educated in ICU by the fourth and fifth authors in two sessions. Some records had incomplete information, we tried to take the information from the staff or families.

## Conclusion

The results of this study showed that the prevalence of oral cavity disorders in patients hospitalized in special care units in the Ilam and Kermanshah provinces was high, and only 5.8% of these patients had the normal oral condition. In patients with less education, older age, unmarried patients, lower levels of consciousness, Ng-tube, and patients who have not brushed their teeth, the overall condition of the mouth was significantly worse. The results of this research can be used by practitioners, nursing education designers, and administrators to adjust the training program for nursing students. Moreover, it is crucial for periodical visits of patients by a dentist in ICUs. The nursing students also should be familiar with the concepts of oral care and disorders in ICUs. It is recommended to use the results of the present research for the prevention, treatment, and reduction of oral complications in ICUs.

## Data Availability

The datasets generated and analyzed during the current study are not publicly available due to our institution’s regulations but are available from the corresponding author on reasonable request.

## Abbreviations

ICU: Intensive care unit
VAP: Ventilator-dependent pneumonia
BOAS: Beck Oral Assessment Scale
NG: Naso-gastric
